# Colorectal cancer ascertainment through cancer registries, hospital episode statistics, and self-reporting compared to confirmation by clinician: A cohort study nested within the UK Collaborative Trial of Ovarian Cancer Screening (UKCTOCS)

**DOI:** 10.1016/j.canep.2018.11.011

**Published:** 2019-02

**Authors:** Darren S. Thomas, Aleksandra Gentry-Maharaj, Andy Ryan, Evangelia-Ourania Fourkala, Sophia Apostolidou, Matthew Burnell, Wendy Alderton, Julie Barnes, John F. Timms, Usha Menon

**Affiliations:** a Women’s Cancer, Institute for Women’s Health, University College London, London WC1E 6BT, UK; b MRC Clinical Trials Unit at UCL, Institute of Clinical Trials & Methodology, University College London, London WC1V 6LJ, UK; c Abcodia Ltd., UK

**Keywords:** Colorectal neoplasms, Electronic health records, Registries, Death certificates, Hospital episode statistics, Self report

## Abstract

**Background:**

Electronic health records are frequently used for cancer epidemiology. We report on their quality for ascertaining colorectal cancer (CRC) in UK women.

**Methods:**

Population-based, retrospective cohort study nested within the UK Collaborative Trial of Ovarian Cancer Screening (UKCTOCS). Postmenopausal women aged 50–74 who were diagnosed with CRC during 2001–11 following randomisation to the UKCTOCS were identified and their diagnosis confirmed with their treating clinician. The sensitivity and positive predictive value (PPV) of cancer and death registries, hospital episode statistics, and self-reporting were calculated by pairwise comparisons to the treating clinician’s confirmation, while specificity and negative predictive value were estimated relative to expected cases.

**Results:**

Notification of CRC events were received for 1,085 women as of 24 May 2011. Responses were received from 61% (660/1,085) of clinicians contacted. Nineteen women were excluded (18 no diagnosis date, one diagnosed after cut-off). Of the 641 eligible, 514 had CRC, 24 had a benign polyp, and 103 had neither diagnosis. The sensitivity of cancer registrations at one- and six-years post-diagnosis was 92 (95% CI 90–94) and 99% (97–100), respectively, with a PPV of 95% (95% CI 92/93–97). The sensitivity & PPV of cancer registrations (at one-year post-diagnosis) & hospital episode statistics combined were 98 (96–99) and 92% (89–94), respectively.

**Conclusions:**

Cancer and death registrations in the UK are a reliable resource for CRC ascertainment in women. Hospital episode statistics can supplement delays in cancer registration. Self-reporting seems less reliable.

## Highlights

• Ninety-two & 99% of colorectal cancers were registered after one & six years.
• Hospital Episode Statistics tend to capture events unregistered after one year.
• Self-reporting by women aged ≥50 was less reliable and had low respondents.
• Electronic health records in the UK are suitable for studying colorectal cancer in women.

## 1 Background

Electronic health records (EHRs) are datasets created for routine administrative purposes. Increasingly, however, they are used to assess health outcomes in large observational studies and randomised controlled trials [[1], [2], [3], [4], [5]]. Evaluating their quality is therefore crucial.

National registries are responsible for cancer registration (CR) in Northern Ireland, Scotland, and Wales, while cancers diagnosed in England are registered by one of eight regional hubs. English registries have been shown by one-directional comparisons to capture 98% of colorectal cancers (CRCs) recorded in routine healthcare databases [6,7]. However, such estimates are likely overestimated since it is unlikely for an individual database to have complete coverage of all events, while iterative refinements to the registration process require ongoing evaluations.

Hospital episode statistics (HES) is an administrative dataset that documents all admissions and attendances to NHS Trusts in England. Its secondary uses for research has been reviewed [8]. HES is appealing since the compulsory recording of hospital events, each year, amasses ∼125-million diagnostic and procedural records amenable to digital analyses [9], while their coding from case notes by dedicated professionals is generally accurate [10,11]. Despite the extensive coverage of CRC events recorded in HES [12], its reliability has not been determined.

Self-reporting (SR), meanwhile, remains an option for identifying events where EHRs are unavailable or inaccessible. However, the reliability of self-reporting is currently unclear, particularly in spite of increased detection of adenomatous polyps [13] and potential confusion resulting from repeat testing due to technical issues and false positive screening tests [14].

We herein determined the feasibility of using EHRs and self-reporting for CRC ascertainment in UK women. We report on the sensitivity and positive predictive value (PPV) of cancer and death registrations, HES, and self-reporting relative to treating clinician’s confirmation. We also explore the reliability of self-reporting of CRC and the factors that determine the accurate self-reporting of CRC.

## 2 Methods

### 2.1 Study design

The present retrospective cohort study was nested within the UK Collaborative Trial of Ovarian Screening (UKCTOCS). During 2001–05, 202,638 postmenopausal women aged 50–74 were randomised to UKCTOCS trial centres across England, Northern Ireland, and Wales following an invitation from health authorities [15]. All were asked to provide their ethnicity, postcode, height, and weight by postal questionnaire at recruitment. Postcodes were updated throughout the trial. The study cohort were women who were identified as having been diagnosed with CRC since randomisation (see *Data sources*) and gave consent for access to their medical records (see *Clinician’s confirmation*).

### 2.2 Data sources

#### 2.2.1 Electronic health records

Women were linked to their EHRs by NHS number. CRs were up-to-date until 19 May 2011 for England & Wales and 23 February 2010 for Northern Ireland. DCs were up-to-date until 19 May 2011 for England & Wales and 9 March 2011 for Northern Ireland. In- and outpatient HES were received for those in England from 2001 and 2003, respectively, until 22 July 2010. CR, DC, and HES records were reviewed for malignant neoplasms of the colon or rectum (ICD10 C18–C20) diagnosed following randomisation to the UKCTCOS. Duration of follow-up was calculated from the date of randomisation to the date of the latest update (or date of loss to follow-up if before).

#### 2.2.2 Postal questionnaire

All participants were able to self-report CRC using postal questionnaires first sent at 3–5-years post-randomisation (FUQ I) and thereafter in April 2014 (FUQ II) [15]. Each questionnaire included an item on whether the woman had been diagnosed with ‘bowel/colorectal cancer’. FUQ I also requested data on their education (college/university degree or nursing/teaching qualification or A-/O-level or vocational qualification or other or none of the above) alcohol use (how many units consumed on average per week), and smoking habit (never/ever).

#### 2.2.3 Trial database

Incidental notifications were received via the UKCTOCS staff due to 1) investigation of a possible ovarian cancer diagnosis after a positive screening result or 2) a participant citing colorectal cancer diagnosis as a reason for withdrawal from the trial. Half of all UKCTOCS women were screened via annual serum CA125 (25%) or transvaginal ultrasound (25%)**,** while the remaining half were controls who received no screening.

### 2.3 Clinician’s confirmation

All women with a possible CRC diagnosis was identified on 24 May 2011. In May 2012, a postal questionnaire (CRCQ) was sent to the treating clinician (GP by default or treating hospital consultant if self-reported in the FUQ I), requesting the diagnosis date, primary site, stage, grade, morphology, and treatment details (Supplementary Fig. S1). The CRCQ requested details of a specialist if the initial contact was unable to provide complete data, who was contacted if necessary. Reminder CRCQs were sent to non-responding clinicians. Questionnaire outcomes (confirmed CRC, benign polyp, no CRC or benign polyp) were captured in the UKCTOCS Trial Management System. Where multiple CRCQs per women were obtained, a confirmed CRC superseded one which reported a benign diagnosis. The earliest of two cancer diagnosis dates was used where necessary. Cancers without a diagnosis date and those diagnosed after 24 May 2011 were excluded.

### 2.4 Data analyses

#### 2.4.1 Non-response bias

The likelihood of a non-response bias was accessed by multivariable analysis (Kruskal-Wallis/Fisher’s Exact & post-hoc pairwise Wilcox rank sum/χ2 with Bonferroni adjustment) of the characteristics and composition of notifications for women with a clinician's confiration and those whose clinician did not respond.

#### 2.4.2 Sensitivity & PPV

The number of true positive (TP), true negative (TN), false positive (FP), and false negatives (FN) notifications for each dataset were determined by pairwise comparison to clinician’s confirmation (gold-standard). The sensitivity (TP/(TP + FN)), PPV (TP/(TP + FP)), and 95% confidence intervals were then computed for each data source. The CR analysis included events diagnosed ≥ one year before the latest registry update (19 May 2010 for England & Wales and 23 February 2009 for Northern Ireland). The DC analysis included only cancers with death dates before the latest update (19 May 2011 for England & Wales or 9 March 2011 for Northern Ireland). For HES, only women from English centres with a diagnosis date before 22 July 2010 (HES update 22 July 2010) were included. Our SR analysis included women who had a diagnosis date before the date their FUQ I or II was returned. The use of EHRs and SR to complement delays in CRs was also assessed via comparison of the adjunct sensitivities and PPVs for CRCs diagnosed ≥1 year in advance of latest registry update. Adjunct analyses of CR & HES and CR & DC & HES were restricted to England.

#### 2.4.3 Timeliness and completeness

We assessed the completeness of CRs by determining the proportion (%) of all confirmed cancers that were registered in updates received by 4 September 2016 for England & Wales and 15 April 2015 for Northern Ireland. Curation times for CRs were defined as the years between diagnosis and latest update.

#### 2.4.4 Specificity & NPV

Specificities and negative predictive values (NPVs) were estimated relative to the expected number of cases derived from an age-standardised rate of 57.2 cases per 100,000 person-years [16] and total years of follow-up. Full details are disclosed in Supplementary Method M1.

#### 2.4.5 Determinants of accurate self-reporting

A binomial logistic regression model was fitted to identify the variables predictive for self-reporting concordant with the clinician’s confirmation. The analysis was restricted to those who returned their FUQ. Outcomes were either concordant (TP & TN) or discordant (FP & FN) self-reporting. Predictive determinants were age (at self-report), BMI (at recruitment to the UKCTOCS), education (low: A-/O-level or vocational qualification; high: college/university degree or nursing/teaching qualification; other: none of the above), alcohol use (non-drinker/<1 unit per day/≥1 unit per day), smoking habit (never/ever), and socioeconomic status (Index of Multiple Deprivation 2015 (IMD) derived from postcode (England only)). The IMD is a composite measure of seven socioeconomic indicators that stratifies all English postcodes on a gradient from most to least deprived [17]. Outliers (>3 SD of the mean/± 1.5 IQR) were entered as missing. Missing data were imputed five times using predictive mean matching. Logit coefficients for each imputed dataset were pooled according to Rubin’s rules [18]. Odds ratios were adjusted for all variables listed.

#### 2.4.6 Statistical analyses

Five-way Venn diagrams were produced using the online InteractiVenn tool [19]. All other analyses were made with R version 3.3.2 [20] running the epiR, ggplot2, mice, and outliers packages. A *p* value < 0.05 was considered significant.

## 3 Results

**Fig. 1:**
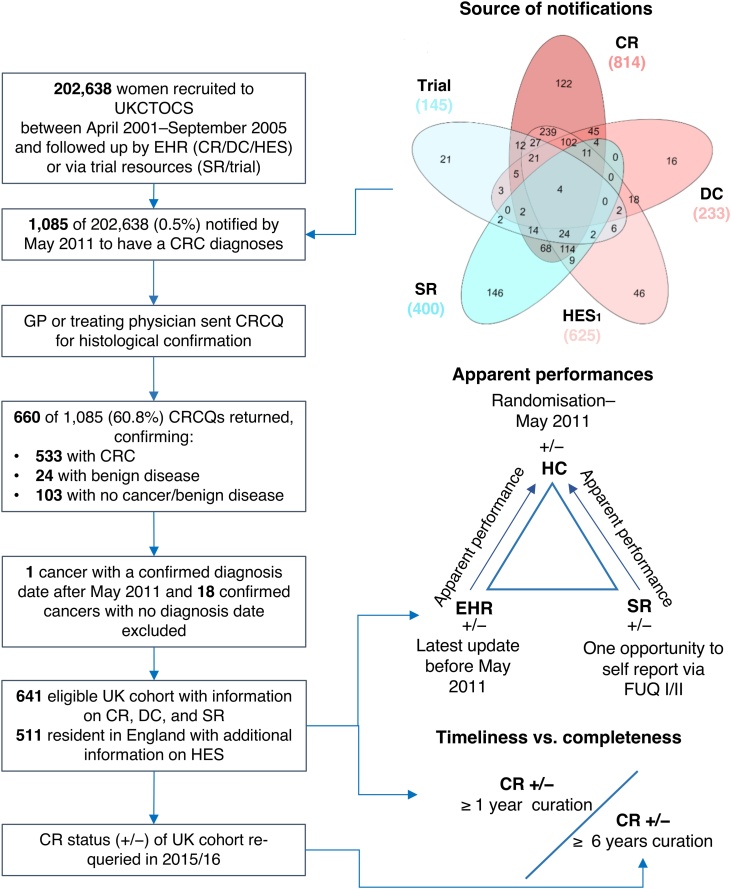
**Study overview.** All women enrolled in the UKCTOCS were monitored for CRCs diagnosed since being randomised via linkage to electronic health records, self-reporting, and by information discerned via the trial itself. The treating clinician or general practitioner of each woman notified to us were contacted for clinician’s confirmation of all events. Apparent performances of electronic health records and self-reporting were assessed against the outcome of clinician’s confirmation. Completeness of cancer registrations at 19 May 2010 (England & Wales)/ 23 February 2009 (Northern Ireland) was compared to those received by 04 September 2016 (England & Wales)/ 15 April 2015 (Northern Ireland). 1 England only. Abbreviations: UKCTOCS, UK Collaborative Trial of Ovarian Cancer Screening; CR, cancer registration; DC, death certification; HES, Hospital Episode Statistics; SR, self-reporting; CRCQ, colorectal cancer questionnaire; HC, clinician’s confirmation; GP, general practitioner; IMD, Index of Multiple Deprivation; EHR, electronic health records (CR, DC, HES).

We received 2,217 notifications of a post-randomisation CRC diagnosis for 1,085 women (Fig. 1). These included 814 CRs, 233 DCs, 625 HES, 400 SRs (FUQ I only), and 145 notifications via the trial database. Clinicians of all 1,085 women were sent a CRCQ. Responses were received from 660 (61%). Nineteen women were excluded as 18 of the confirmed cancers had no diagnosis date on the returned CRCQ and one cancer was diagnosed after May 2011 (self-reported in 2006). Overall, 641 women were eligible for analysis. Of these, 514 had a verified CRC, 24 had a benign polyp, while 103 had never been diagnosed with malignant or benign colorectal disease. Histology reports were disclosed for 15% (75/514) of verified CRCs. There were 1,173 TP notifications and 152 FP notifications (38 for benign disease, 114 for no malignant/benign diagnosis).

The contribution of each notification to the pools of TPs and FPs is presented in Supplementary Fig. S2. Cancer registrations contributed to 38.6% (453) of 1,173 TP notifications, followed by HES (27.9% (327)), self-reporting (18.0% (211)), death certificates (8.4% (98)), and trial resources (7.2% (84)). CRs provided the greatest contribution of uniquely recorded TPs (4.6% (54)), while SR (0.7% (8)) and DC (0.3% (4)) provided few. Overlap in TP events between two datasets ranked in order of CR & HES (38.2% (298/780)), CR & SR (29.1% (193/664)), HES & SR (23.8% (128/538)), DC & HES (16.2% (69/425)), CR & DC (15.8% (87/551)), and DC & SR (2.6% (8/309)) (Supplementary Fig. S2a). Contributions to the pool of 152 FP notifications ranked in order of SR (62.5% (95)), CR (16.4% (25)), HES (14.5% (22)), trial resources (5.3% (8)), and DC (1.3% (2)) (Supplementary Fig. S2b). Notably, 58.6% (89) of FPs were uniquely notified by SR—eight where a benign adenoma was present and 84 where no benign/malignant diagnoses were verified.

Baseline characteristics are presented in Table 1 (& further stratified in Supplementary Table S1). Women whose clinicians did not respond (NR) had a greater proportion of notifications informed by DC (15%) to TPs (8%) and FPs (1%). NRs and TPs were similar but differed to FPs in their proportion of notifications informed by CR (37, 39%, & 16%, respectively), HES notifications (26, 28, and 14%), and SR (16, 18, & 62%). NRs were comparable to TPs in their age, BMI, ethnicity, education, alcohol use, and smoking habit and differed to FPs in age, IMD score, education, alcohol use, and smoking habit (Table 1). NRs had a markedly greater proportion of deaths (38%) than FPs (5%) and TPs (24%).

**Table 1:** Non-responders show notable similarities to True Positives and differences to False Positives.

	Cohort			*p* value^a^
	Responders (TP)	Responders (FP)	Non-responders (NR)	
	Median (Range)			
Age	72 (57–83)	68 (57–82)	72 (57–83)	NR vs. FP (0.018)
BMI (Kg m-^2^)	25.8 (10.3–110.8)	26.4 (18.7–150.0)	25.6 (0.1–47.2)	0.753
IMD score	13.6 (1.6–73.9)	11.4 (2.5–74.4)	16.4 (1.6–70.8)	NR vs. TP (0.011) & FP (0.003)

	Count (%)			
Cohort size	514 (100)	127 (100)	425 (100)	
Alcohol				NR vs. FP (< 0.001)
Non-drinker	83 (16)	16 (13)	71 (17)	
< 1 unit a day	178 (35)	67 (53)	124 (29)	
≥ 1 unit a day	71 (14)	37 (29)	55 (13)	
Missing	182 (35)	7 (6)	175 (41)	

Deaths^b^				NR Vs. TP (< 0.001) & FP (< 0.001)
Alive	390 (76)	121 (95)	263 (62)	
Deceased	124 (24)	6 (5)	162 (38)	

Education				NR vs. FP (< 0.001)
Low	131 (25)	50 (39)	104 (24)	
High	89 (17)	43 (34)	54 (13)	
Other	106 (21)	25 (20)	95 (22)	
Missing	188 (37)	9 (7)	172 (40)	

Ethnicity				0.821
White	493 (96)	124 (98)	411 (97)	
Black	9 (2)	2 (2)	7 (2)	
Other	9 (2)	1 (1)	3 (1)	
Missing	3 (1)	0 (0)	4 (1)	

Smoking				NR vs. FP (< 0.001)
Ever	109 (21)	43 (34)	120 (28)	
Never	187 (36)	64 (50)	135 (32)	
Missing	218 (42)	20 (16)	170 (40)	

Notifications	1,173 (100)	152 (100)	841 (100)	NR vs. TP (0.001) & FP (< 0.001)
CR	453 (39)	25 (16)	312 (37)	
DC	98 (8)	2 (1)	125 (15)	
HES	327 (28)	22 (14)	220 (26)	
SR	211 (18)	95 (62)	137 (16)	
Trial	84 (7)	8 (5)	47 (6)	

a *p* values refer to the multivariable test (Kruskal-Wallis/Fisher’s Exact) if no significance detected or the Bonferroni-adjusted, pairwise post-hoc test(s) (pairwise Wilcox rank sum/χ^2^) if significance detected.

b at clinician’s confirmation. Abbreviations: TP; True Positives (Responders); FP, False Positives (Responders); NR, Non-responders; CRCQ, Colorectal Cancer Questionnaire.

### 3.1 Cancer registrations

The sensitivity and PPV were estimated from 618 verified women (491 with CRC diagnosed one year before the latest update, 24 with a benign polyp, 103 with no colorectal disease). Median follow-up from randomisation to registry update or loss to follow-up was 6.5 years (IQR 2.1; n 618). Curation times ranged from 1.0 to 9.1 years (median 4.1; IQR 3.2; n 491).

CR notified of 54 unique TP events not captured by other sources. There were 38 CRCs without a CR after a minimum of one-year curation. Of these, 32 were registered when curation was extended to 6.3–14.4 years (median 9.4; IQR 3.2), while six residents in England remained unregistered after 6.8, 7.5, 10.5, 11.5, 13.1, and 13.6 years of curation (Table 2b & Supplementary Table S2). Four of the FP registrations (2 benign and 2 no CRC) were rescinded by 2015/16, while 2 TN (1 benign and 1 no CRC) became FP. Overall, a further 36 women were correctly classified (32 CRC TPs and 2 benign & 2 no CRC TNs) and two were incorrectly registered (1 benign & 1 no CRC FPs) when curation was extended.

**Table 2:** Distribution of true and false notifications by data source.

**Fig. 2:**
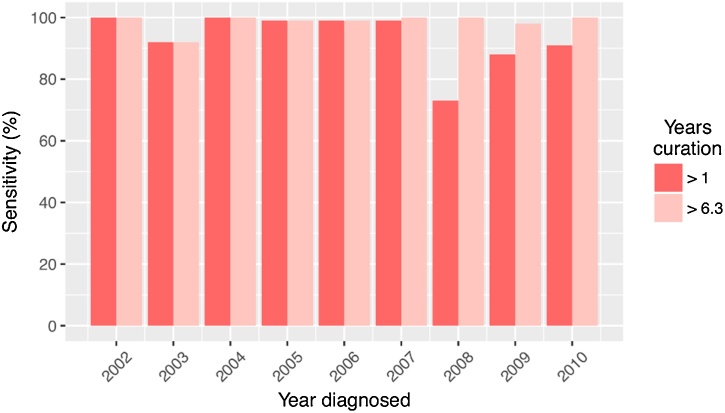
**Sensitivity of cancer registrations curated after a minimum of 1 & 6.3 years.** Sensitivities by year of diagnosis (2002–10 inclusively) were 100, 92, 100, 99, 99, 99, 73, 88, and 91% with allowance of 1.0–9.1 years curation (CR update in 2010/11) and 100, 92, 100, 99, 99, 100, 100, 98, and 100% when supplemented with registrations received until 2015/16 (6.3–14.4 years curation).

The sensitivity and PPV of CR after allowing a minimum of 1 year for their curation were 92% (453/491; 95% CI 90–94) and 95% (453/478; 95% CI 92–97), respectively (Table 3). When supplemented with registrations received until 2015/16, the sensitivity of CR increased to 99% (485/491; 95% CI 97–100), while the PPV remained at 95% (485/508; 95% CI 93–97). Sensitivities by year of diagnosis (2002–2010 inclusively) after a minimum of one or six years of curation are presented in Fig. 2. Specificity and NPV were estimated from 202,230 women. Relative to the 850 cases expected after 1,486,350 person-years follow-up, the specificity and NPV were 100% (201,485/201,515; 95% CI 100–100) and 100% (201,485/201,551; 95% CI 100–100), respectively.

**Table 3:** Performance estimates for electronic health records and self-reporting.

Dataset	Sensitivity^a^ (95% CIs)	PPV^a^ (95% CIs)	Specificity^b^ (95% CIs)	NPV^b^ (95% CIs)
CR^c^	0.92 (0.90–0.94)	0.95 (0.92–0.97)	1.00 (1.00–1.00)	1.00 (1.00–1.00)
CR^d^	0.99 (0.97–1.00)	0.95 (0.93–0.97)	—	—
DC	0.97 (0.92–0.99)	0.98 (0.93–1.00)	0.97 (0.97–0.97)	1.00 (1.00–1.00)
HES	0.82 (0.78–0.86)	0.94 (0.91–0.96)	1.00 (1.00–1.00)	1.00 (1.00–1.00)
SR^d^	0.91 (0.86–0.94)	0.69 (0.63–0.74)	1.00 (1.00–1.00)	1.00 (1.00–1.00)

a relative to clinician’s confirmation.

b relative to expected cases.

c 1–9 years curation (median 4.1, IQR 3.2).

d 6–14 years curation (median 9.4, IQR 3.2). Abbreviations: PPV, positive predictive value; NPV, negative predictive value; CIs, confidence intervals; CR, cancer registration; DC, death certificate; HES, Hospital Episode Statistics; SR, self-reporting.

### 3.2 Death registrations

Sensitivity and PPV were estimated from the 104/641 (16.2%) verified women who died before the latest DC update (102 cancers diagnosed before the latest update, 2 with no cancer/benign (Table 2c)). Median follow-up from randomisation to registry update or loss to follow-up was 4.8 years (IQR 3.2; n 104). The sensitivity and PPV of DCs were 97% (98/102; 95% CI 92–99) and 98% (98/101; 95% CI 93–100), respectively (Table 3). Specificity and NPV were estimated from 7,202 registered deaths. Relative to the 19 cases expected after 33,968 person-years follow-up, the specificity and NPV were 97% (6,968/7,183; 95% CI 97–97) and 100% (6,968/6,969; 95% CI 100–100), respectively.

### 3.3 Hospital episode statistics

The sensitivity and PPV were estimated from 502 verified women who were randomised from centres in England (397 with confirmed CRC diagnosed before 22 July 2010, 21 with a benign polyp, 84 with no colorectal disease (Table 2d)). Median follow-up from randomisation to HES update or loss to follow-up was 6.7 years (IQR 2.2; n 502). Eleven TP HES notifications were unique and were all diagnosed in 2008–09. The sensitivity and PPV for HES notifications were 82% (327/397; 95% CI 78–86) and 94% (327/349; 95% CI 91–96), respectively (Table 3). Specificity and NPV were estimated from 157,839 women in England. Relative to the 616 cases expected after 1,076,512 person-years follow-up, the specificity and NPV were 100% (157,105/157,223; 95% CI 100–100) and 100% (157,105/157,214; 95% CI 100–100), respectively.

### 3.4 Self-reporting

Of 641 eligible women, 353 (55.1%) completed a FUQ (291 FUQ1, 62 FUQ II). Of these, 233 had a confirmed CRC, 22 had a benign polyp, and 98 had no malignant/benign colorectal disease (Table 2e–f). The sensitivity and PPV of SR in the cohort of responders were 91% (211/233; 95% CI 86–94) and 69% (211/306; 95% CI 63–74), respectively (Table 3). The sensitivity where non-responses were negative by default was 41% (211/514; 95% CI 37–45) at a 69% PPV. Specificity and NPV were estimated from 144,313 women who returned their FUQ. Relative to the 321 cases expected after 561,274 person-years follow-up, the specificity and NPV were 100% (143,883/143,992; 95% CI 100–100) and 100% (143,883/143,913; 95% CI 100–100), respectively.

We fitted a logistic regression to ascertain the variables associated with concordant self-reporting. The adjusted ORs are presented in Table 4. Missing data were imputed for IMD (83 (9 England, 22 Northern Ireland, 52 Wales)), smoking (51), education (13), alcohol (8), age at self-report (4), and BMI (3 including 1 outlier). Baseline characteristics for women included in this model are summarised in Supplementary Table S1. Age at self-report markedly increased the odds of being concordant with their clinician’s confirmation (adjusted OR 1.05; 95% CI 1.01–1.10; *p* 0.026). No other associations were statistically significant.

**Table 4:** Variables associated with self-reporting concordant with clinician’s confirmation.

Variable	OR^a^ (95% CIs)	*p* value
(Intercept)	0.22 (0.01–7.53)	0.395
Age (year)	1.05 (1.01–1.10)	0.026*
BMI (Kg/m^2^)	0.98 (0.91–1.05)	0.504
IMD score	1.01 (0.98–1.04)	0.441

Alcohol		
0	1.00	
< 1 U/day	0.48 (0.20–1.13)	0.091
> 1 U/day	0.38 (0.15–1.00)	0.051

Smoking		
Never	1.00	
Ever	1.18 (0.63–2.19)	0.604

Education		
Low	1.00	
High	1.19 (0.60–2.36)	0.613
Other	0.86 (0.41–1.83)	0.698

a Adjusted for all variables listed. n 353. Age at self-report. Abbreviations: OR, odds ratio; BMI, Body Mass Index; IMD, Index of Multiple Deprivation.

### 3.5 Trial database

Notification via the UKCTOCS trial centre accounted for few of the overall TP (7.2%; 84/1,173), FP benign (7.9%; 3/38), and FP no CRC/benign notifications (4.4%; 5/114) but captured 12 events that would have otherwise been missed (Supplementary Fig. S2a). The majority of TPs (84.5% (71/84)) and FPs (87.5% (7/8)) were informed by reasons for withdrawal from the UKCTOCS.

### 3.6 Adjunct datasets

Given the demonstrated delay in cancer registrations, researchers may be interested in how best to supplement their analyses. There were 38 cancers not registered 1 year after diagnosis (36 in England). HES, SR, and DC captured 77.8% (28/36), 36.8% (14/38), and 21.1% (8/38) of these events, respectively. The sensitivity and PPV of CR & HES (n 501) were 98% (388/396; 95% CI 96–99) and 92% (388/422; 95% CI 89–94); CR & SR (n 618): 95% (467/491; 95% CI 93–97) and 80% (467/581; 95% CI 77–84); and CR & DC (n 618): 94% (461/491; 95% CI 91–96) and 95% (461/487; 95% CI 92–96). The sensitivity and PPV of CR, DC & HES combined (n 501) were 98% (388/396; 95% CI 96–99) and 92% (388/422; 95% CI 89–94).

## 4 Discussion

Advances in healthcare are achieved through high-quality epidemiological studies informed by a comprehensive and reliable ascertainment of outcomes. We have herein evaluated the performance of EHRs in addition to self-reporting for ascertaining diagnoses of CRC in UK women in comparison to a patient’s clinical records. We found that 92% of CRCs diagnosed in the UK during 2001–10 were registered within one year, and 99% within six years. Researchers looking to overcome delays in the curation of cancer registrations are advised to use HES in adjunct, which combined had a sensitivity of 98% and a PPV of 92%. Finally, self-reporting of CRC for standalone or adjunct ascertainment was not useful owing to high false positivity and low response rates.

Registration of cancers by regional registries are the cornerstone of national and international cancer surveillance [21] and have hitherto informed the implementation of CRC screening programmes [1,2]. An incomplete ascertainment of events, however, can skew analyses. Nonetheless, our group previously concluded that incomplete registration of CRC is unlikely as 85% of self-reported (but not verified) CRCs were registered within five years of diagnosis [22]. The higher sensitivity of CRC cancer registration reported here (92% vs 85%) is likely due to our exclusion of previously unknown false positive self-reported CRCs. Elsewhere, it is reported that 98% of surgically treated CRCs recorded in HES during 2001–07 were registered [6], while 98% of primary care records were captured within four years [7].

We found no issues with the reliability of HES (PPV 94%). This is in alignment with a recent meta-analysis [10]. HES did, however, have limited sensitivity that would likely preclude its use for standalone ascertainment. These missed cases are likely due to coding errors, emergency admissions, death certificate-only registrations, or privately-treated patients. Nonetheless, a similar sensitivity (83%) was reported for prostate cancers recorded by HES relative to medical notes [23].

Self-reporting of CRC by post was unreliable. While the sensitivity (91%) of responders was similar to CR at one year (92%), a low response rate (55%) limited the sensitivity to 41%. Furthermore, the PPV was 69%, and some women misreported their benign polyp as cancer. Studies in the USA have reported similar sensitivities (83–85%) for postal self-report, albeit at a lower PPV (54%) [24,25]. Our findings align with reports that British interviewees markedly under-report CRC diagnoses in close relatives [26], but contrasts in that they reliably self-report their participation in CRC screening [27]. As screening becomes widely accepted, an ongoing evaluation of how it might influence the layperson’s ability to reliably self-report would be insightful.

Our study has several limitations. Firstly, we did not verify the absence of cancer in those without a notification, and thus the reliability of the sensitivity estimates are dependent on the number of false negatives missed. Secondly, the potential for bias in the 39% of non-responding clinicians contacted should not be overlooked for an underascertainment of FPs through non-response would overstate the PPV, while an underascertainment of TPs would underestimate the sensitivity. Nonetheless, barring a higher proportion of mortality at point of clinician contact and notifications via death certificate, non-responders were in closer alignment to TPs than FPs in the notifications received, and we suspect the risk of bias to be minimal. Next are issues of generalisability. Men are disproportionally affected by CRC [16] while also being less likely to undertake FOBT screening than women [28], but were not studied here. Ethnic minorities, too, are less likely to undertake screening [28] but were underrepresented in our cohort. Finally, our findings may be affected by ‘healthy-volunteer’ bias that typically affects those willing to enrol in trials [29], while it would also be reasonable to assume that recall biases arising from past diagnoses of CRC in the family or prior colonoscopy for suspected cancer could aid concordant self-reporting and would, ideally, be accounted for in analyses if obtained.

Weaknesses notwithstanding, our study updates the current performance estimates of colorectal cancer registrations [6,7]. Our estimates are reliable though us studying verified events identified via myriad routes. It is strengthened further from the high rate at which patients’ EHRs were linked.

## 5 Conclusions

Electronic health records in England, Northern Ireland & Wales are a reliable resource for ascertaining colorectal cancer events in women. Researchers looking to supplement delays in the registration of CRCs should use hospital episode statistics in adjunct. Self-reporting of colorectal cancer by women is neither reliable nor is it useful in adjunct with electronic health records.

## Authorship contribution

DST, AG-M, AR, MB, and UM contributed to the design of the study. DST prepared and analysed the data and created all tables and figures. DST, AG-M, and UM drafted and revised the manuscript. All authors contributed to the interpretation of data and revision of drafts. All approved the final version to be published.

## Competing interests

UM has stock ownership in and has received research funding from Abcodia Ltd. UM has received grants from the Medical Research Council, Cancer Research UK, the National Institute for Health Research, and The Eve Appeal.

## Ethics approval

National Research Ethics Service Committee of East Midlands, Derby [REC 13/EM/0191]. The UKCTOCS was approved by the UK North West Medical Research Ethics Service [MREC 00/8/34].

## Patient consent

All subjects gave their written, informed consent for researchers to access their medical notes, for their NHS number to be linked to external datasets, and for their anonymised data to be used in secondary studies.

## Funding

The present study was supported by the Medical Research Council[Industrial CASE Studentship: MR/J006718/1]. The Medical Research Council had no involvement in design, undertaking, or reporting of the study. UM was supported by the National Institute for Health Research, Biomedical Research Centre, University College London Hospital. The UKCTOCS was funded by the Medical Research Council, Cancer Research UK, the Department of Health, and The Eve Appeal.
