# Supplementary material for: Colorectal cancer ascertainment through cancer registries, hospital episode statistics, and self-reporting compared to confirmation by clinician: A cohort study nested within the UK Collaborative Trial of Ovarian Cancer Screening (UKCTOCS)
Source: Cancer Epidemiol. 2019 Feb;58:167–74. doi: 10.1016/j.canep.2018.11.011 (PMC6363963; doi:10.1016/j.canep.2018.11.011)
Supplement: Supplementary file 1 [file mmc1.pdf]

SUPPLEENTARY METHOD M1

Specificity & NPV.

Specificities and NPVs were estimated relative to the expected number of cases derived from an age-standardised rate of 57.2 cases per 100,000 person years [1] applied to the total years of follow-up for subsets of all 202,365 consenting women who 1) lived in the UK (CR analysis), 2) had died before the latest DC update (DC analysis), 3) lived in England (HES analysis), and 4) who returned their FUQ before 24 May 2011 (SR analysis). Total years of follow-up was the sum of all years followed-up from randomisation to latest update (see *Electronic health records*), or date of death if before. Column totals of the confusion matrix were inferred from the expected number of cases (TP+FN) or sample size minus expected cases (FP+TN). Row totals of the confusion matrix were inferred from the number of notifications (TP+FP) or sample size minus number of notifications (FN+TN). Using the sensitivity estimates (Table 3 or Supplementary Table M1 for non-rounded, crude estimates), the number of TPs can be calculated as 1) sensitivity * (TP+FN). Individual cells (i.e. FN, TP & TN) were then deduced from subtraction of TP from column/row totals. The specificity (TN/(FP+TN)), NPV (TN/(FN+TN)), and 95% confidence intervals were then computed.

SUPPLEMNETARY TABLE M1: Crude sensitivities and PPVs

Dataset	Crude sensitivity	Crude PPV
CR_1	0.9226069	0.9476987
DC	0.9702970	0.9800000
HES	0.8236776	0.9369628
SR	0.9055794	0.6895425

[1] 1–9 years curation (median 4.1, IQR 3.2). Abbreviations: CIs, confidence intervals; PPV, positive predictive value; CR, cancer registration; DC, death certificate; HES, Hospital Episode Statistics; SR, self-reporting.

Example: Cancer registrations

There were 814 (+) cancer registrations received by latest registry update and 850 expected cancers:

TP + FN = 850 expected cancers.

FP + TN = 201,515 (202,365 consenting women followed-up minus 850 expected cancers).

TP + FP = 814 (+) cancer registrations received by latest update.

FN + TN = 201,551 (202,365 consenting women followed-up minus 814 (+) cancer registrations).

Since sensitivity of cancer registrations relative to clinical confirmations was 0.9226069, the number of TPs can be calculated as sensitivity * (TP + FN), or 0.9226069 * 850, which equals 784. FN is therefore 66 (850 minus 784), FP is 30 (814 minus 784), and TN is 201,485 (201,515 minus 30 or 201,551 minus 66).

**Confusion matrix based on cancer registrations received and expected number of cases.**			
	**Expected +**	**Expected –**	**Total**
**Notification +**	784	30	**814**
**Notification –**	66	201,485	**201,551**
**Total**	**850**	**201,515**	**202,365**

Example: Death registrations

There were 233 (+) death registrations received by latest registry update and 19 expected cancers:

TP + FN = 19 expected cancers.

FP + TN = 7,183 (7,202 women deceased minus 19 expected cancers).

TP + FP = 233 (+) death registrations received by latest update.

FN + TN = 6,969 (2,202 deceased women minus 233 (+) death registrations).

Since sensitivity of death registrations relative to clinical confirmations was 0.9702970, the number of TPs can be calculated as sensitivity * (TP + FN), or 0.9702970 * 19, which equals 18. FN is therefore 1 (19 minus 18), FP is 215 (233 minus 18), and TN is 6,968 (7,183 minus 215 or 6,969 minus 1).

Confusion matrix based on death registrations received and expected number of cases.			
	**Expected +**	**Expected –**	**Total**
**Notification +**	18	215	**233**
**Notification –**	1	6,968	**6,969**
**Total**	**19**	**7,183**	**7,202**

Example: Hospital Episode Statistics

There were 625 (+) Hospital Episode Statistics received by latest update and 616 expected cancers:

TP + FN = 616 expected cancers.

FP + TN = 157,223 (157,839 women followed-up minus 616 expected cancers).

TP + FP = 625 (+) Hospital Episode Statistics received by latest update.

FN + TN = 157,214 (157,839 women followed-up minus 625 (+) Hospital Episode Statistics).

Since sensitivity of Hospital Episode Statistics relative to clinical confirmations was 0.8236776, the number of TPs can be calculated as sensitivity * (TP + FN), or 0.8236776 * 616, which equals 507. FN is therefore 109 (616 minus 507), FP is 118 (625 minus 507), and TN is 157,105 (157,223 minus 118 or 157,214 minus 109).

Confusion matrix based on Hospital Episode Statistics received and expected number of cases.			
	**Expected +**	**Expected –**	**Total**
**Notification +**	507	118	**625**
**Notification –**	109	157,105	**157,214**
**Total**	**616**	**157,223**	**157,839**

Example: Self-reporting

There were 400 (+) self-reportings received and 321 expected cancers:

TP + FN = 321 expected cancers.

FP + TN = 143,992 (144,313 women followed-up minus 321 expected cancers).

TP + FP = 400 (+) self-reportings received.

FN + TN = 143,913 (144,313 women followed-up minus 400 (+) self-reportings).

Since sensitivity of Hospital Episode Statistics relative to clinical confirmations was 0.9055794, the number of TPs can be calculated as sensitivity * (TP + FN), or 0.9055794 * 321, which equals 291. FN is therefore 30 (321 minus 291), FP is 109 (400 minus 291), and TN is 143,883 (143,992 minus 109 or 143,913 minus 30).

**Confusion matrix based on self-reportings received and expected number of cases.**			
	**Expected +**	**Expected –**	**Total**
**Notification +**	291	109	**400**
**Notification –**	30	143,883	**143,913**
**Total**	**321**	**143,992**	**144,313**

REFERENCES

[1] Cancer Research UK, Bowel cancer incidence statistics [UK, 2015, ICD-10 C18–C20], (2018). http://www.cancerresearchuk.org/health-professional/cancer-statistics/statistics-by-cancer-type/bowel-cancer/incidence (accessed February 28, 2018).

SUPPLEMENTARY TABLE S1: Baseline characteristics of study cohorts			
	Cohort		
	UK	England	Logistic model
	Median (Range)		
**Age**	71 (57–83)	71 (57–83)	70 (57–83)
**BMI (Kg m^{-2})**	26.0 (10.3–110.8)	26.0 (10.3–110.8)	25.8 (18.3–62.7)
**IMD score**	13.0 (1.6–74.4)	13.0 (1.6–74.4)	12.0 (1.7–74.4)
	Count (%)		
**Cohort size**	**641 (100)**	**511 (100)**	**353 (100)**
**Histological classification**			
CRC	514 (80.2)	406 (79.5)	233 (66.0)
Benign adenoma	24 (3.7)	21 (4.1)	22 (6.2)
No CRC or benign adenoma	103 (16.1)	84 (16.4)	98 (27.8)
**Ethnicity**			
White	617 (96.3)	488 (95.5)	344 (97.5)
Black	11 (1.7)	10 (2.0)	6 (1.7)
Other	10 (1.6)	10 (2.0)	2 (0.6)
Missing	3 (0.5)	3 (0.6)	1 (0.3)
**Education**			
Low	181 (28.2)	146 (28.6)	146 (41.4)
High	132 (20.6)	97 (19.0)	107 (30.3)
Other	131 (20.4)	111 (21.7)	87 (24.6)
Missing	197 (30.7)	157 (30.7)	13 (3.7)
**Alcohol**			
Non-drinker	99 (15.4)	74 (14.5)	75 (21.2)
< 1 unit a day	245 (38.2)	198 (38.7)	184 (52.1)
≥ 1 unit a day	108 (16.8)	87 (17.0)	86 (24.4)
Missing	189 (29.5)	152 (29.7)	8 (2.3)
**Tobacco**			
Ever	152 (23.7)	126 (24.7)	111 (31.4)
Never	251 (39.2)	196 (38.4)	191 (54.1)
Missing	238 (27.1)	189 (37.0)	51 (14.4)

SUPPLEMENTARY TABLE S2: Longitudinal evolution of cancer registrations held for the same women.

Confirmed CRC				Benign polyp				No CRC		
**2010/11**	**2015/16**	**Count**		**2010/11**	**2015/16**	**Count**		**2010/11**	**2015/16**	**Count**
+	+	453		+	+	11		+	+	10
-	+	32		-	+	1		-	+	1
+	-	0		+	-	2		+	-	2
-	-	6		-	-	10		-	-	90

+ cancer registration. - no cancer registration. Thirty-two of the women who had a confirmed diagnosis of CRC and who had no cancer registration in 2010/11 were registered by 2015/16. Only six of the 491 confirmed diagnoses had no cancer registration by 2015/16. Of the 25 false-positive registrations held for women who had either a diagnosis of a benign polyp or had no diagnoses at all, four were rescinded and 21 remained in 2016. Two women who were truly classified as negative were subsequently registered by 2016.

UKCTOCS Coordinating Centre
Gynaecological Cancer Research Centre
Institute for Women's Health, UCL
Maple House, 149 Tottenham Court Road
London, W1T 7DN
Fax: 0203 447 2129

Private and Confidential
«ConsultantTitle» «ConsultantFirstName» «ConsultantSurname»
«ConsultantSpeciality»
«HospAddress1»
«HospAddress2»
«HospTown»
«HospPostCode»

Dear «ConsultantTitle» «ConsultantSurname»,

RE: «V_FirstNames» «V_Surname»
DOB: «V_DateOfBirth»
Address : «V_Address1», «V_Address2», «V_Town», «V_PostCode»
NHS number: «V_NHSNumber»
UKCTOCS reference number: «O_Volunteeref»

Mrs «V_Surname» is a participant in the UKCTOCS ovarian cancer screening trial, which is a major national MRC, CRUK, and DoH funded randomised controlled trial involving 202,638 women in the UK. According to the notifications we have received, Mrs «V_Surname» has been diagnosed with COLORECTAL CANCER. In order to fully utilise the serum samples that Mrs «V_Surname» has donated to cancer research we kindly ask you to complete the questions below, which relate to the histology at diagnosis and any treatment the patient may have received.

Please could you fill in the following questionnaire and send us a copy of the histology if available. A free post envelope is enclosed. I have enclosed a copy of Mrs «V_Surname»'s consent form to take part in the UKCTOCS which provides permission for access to her medical records.

If Mrs «V_Surname» was not under your care, it would be appreciated if you provide the contact details of the consultant responsible for Mrs «V_Surname»'s cancer treatment.

Name of consultant:………………………………………………………………………………..

Address:………………………………………………………………………………………………….

……

Yours sincerely,

Professor Usha Menon

Name of patient: «V_FirstNames» «V_Surname» **DOB: «V_DateOfBirth»**
UKCTOCS reference number: «V_VolunteerRef» **NHS number: «V_NHSNumber»**

SUPPLEMNETARY FIGURE S1: Page One of Colorectal cancer questionnaire (CRCQ) sent to all treating clinicians requesting confirmation and histology reports.

United Kingdom Collaborative Trial of Ovarian Cancer Screening

Date of Diagnosis: _ _/ _ _/ _ _ _ _ (DD/MM/YYYY)

Primary Tumour Site:

☐ Ascending Colon ☐ Transverse Colon ☐ Descending Colon

☐ Sigmoid Colon ☐ Rectum ☐ Caecum ☐ Other (please specify)

Staging Type and Figures:

Stage:

T: N: M:

Dukes:

□ A □ B

□ C □ D

Site of Metastasis:

Grade:

☐ Grade I (Well Differentiated) ☐ Grade II (Moderately Differentiated)

☐ Grade III (Poorly Differentiated) ☐ Unknown

Morphology:

☐ Adenocarcinoma ☐ Mucinous (colloid) adenocarcinoma

☐ Signet-ring cell carcinoma ☐ Other (please specify)………………………...

Treatment:

1. Surgery: Date: _ _/ _ _/ _ _ _ _ (DD/MM/YYYY)

☐ Local excision or simple polypeptomy ☐ Resection and anastomosis

☐ Resection without anastomosis ☐ Other (please specify) ………………

Tumour Size in cms: ☐ Number of nodes removed/ affected: ☐/☐

2. Chemotherapy: ☐ Yes ☐ No Date: _ _/ _ _/ _ _ _ _ (DD/MM/YYYY)

If, Yes a. ☐ FOLFIRI (5-fluorouracil, leucovorin and irinotecan)

b. ☐ FOLFOX (folinic acid (leucovorin), 5-FU, Oxaliplatin)

c. ☐ Other combination (please tick all drugs applicable)

☐ Folinic Acid ☐ Capecitabine ☐ Irinotecan

☐ Oxaliplatin ☐ Bevacizumab (Avastin) ☐ Cetuximab

☐ Panitumumab ☐ Unknown ☐ Other (please specifiy)……………………...

3. Radiotherapy: ☐ Yes ☐ No ☐ Not known

SUPPLEMNETARY FIGURE S1 (continued): Page Two of Colorectal cancer questionnaire (CRCQ) sent to all treating clinicians requesting confirmation and histology reports.

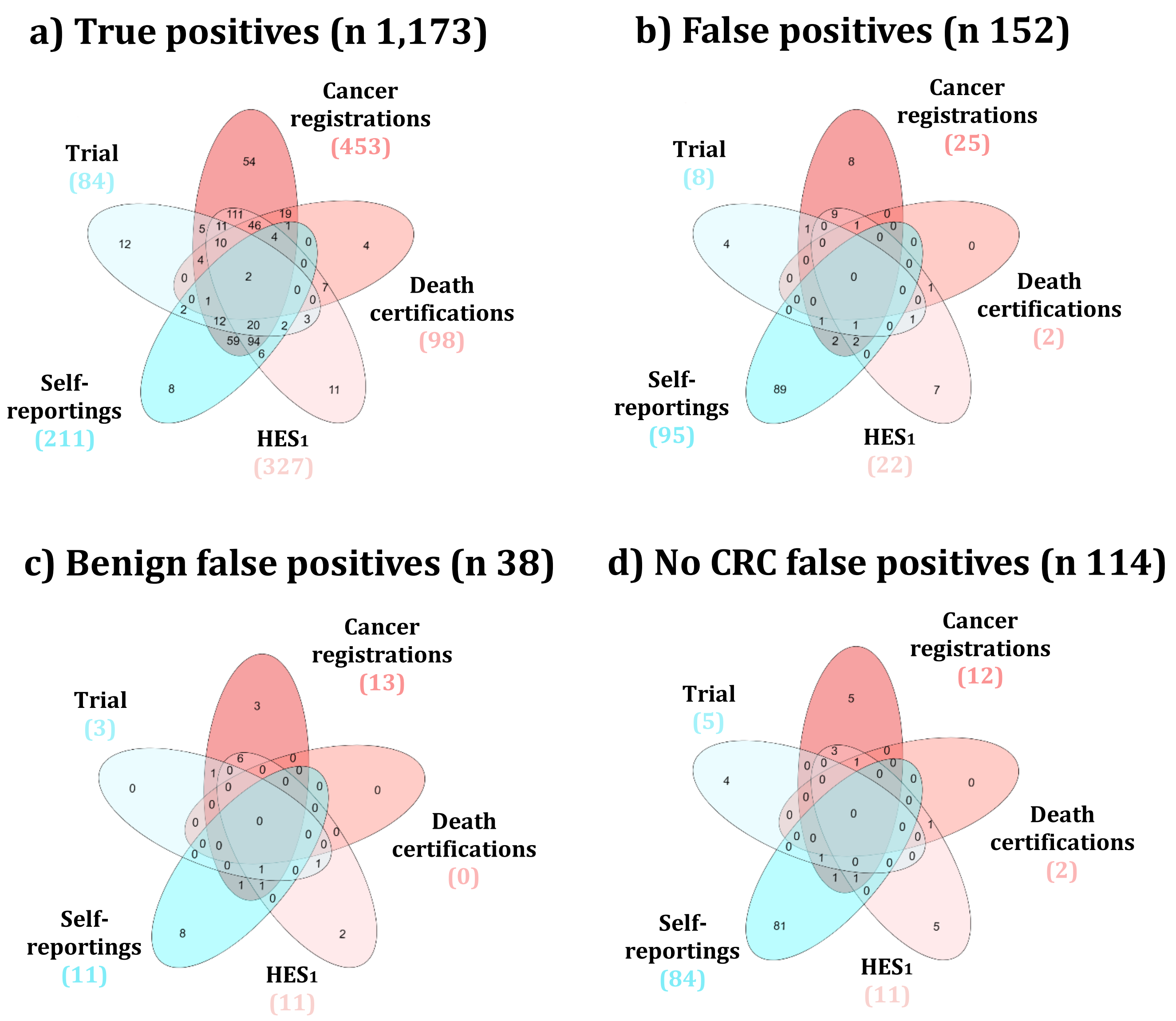

SUPPLEMENTARY FIGURE S2: Distribution of true (a) and false (b–d) positives according to avenue of follow-up. 1 England only. Abbreviations: HES, Hospital Episode Statistics.
